# Author Correction: Transcriptomic and cellular decoding of regional brain vulnerability to neurogenetic disorders

**DOI:** 10.1038/s41467-020-19362-z

**Published:** 2020-11-17

**Authors:** Jakob Seidlitz, Ajay Nadig, Siyuan Liu, Richard A. I. Bethlehem, Petra E. Vértes, Sarah E. Morgan, František Váša, Rafael Romero-Garcia, François M. Lalonde, Liv S. Clasen, Jonathan D. Blumenthal, Casey Paquola, Boris Bernhardt, Konrad Wagstyl, Damon Polioudakis, Luis de la Torre-Ubieta, Daniel H. Geschwind, Joan C. Han, Nancy R. Lee, Declan G. Murphy, Edward T. Bullmore, Armin Raznahan

**Affiliations:** 1 Developmental Neurogenomics Unit, National Institute of Mental Health, Bethesda, MD USA; 2 Department of Psychiatry, University of Cambridge, Cambridge, UK; 3 School of Mathematical Sciences, Queen Mary University of London, London, UK; 4 The Alan Turing Institute, London, UK; 5 McConnell Brain Imaging Centre, Montreal Neurological Institute and Hospital, Montreal, QC Canada; 6 McGill Centre for Integrative Neuroscience, McGill University, Montreal, QC Canada; 7 Department of Neurology, Center for Autism Research and Treatment, Semel Institute, David Geffen School of Medicine, UCLA, Los Angeles, CA USA; 8 Department of Psychiatry and Biobehavioral Sciences, Semel Institute, David Geffen School of Medicine, UCLA, Los Angeles, CA USA; 9 Department of Human Genetics, David Geffen School of Medicine, UCLA, Los Angeles, CA USA; 10 Departments of Pediatrics and Physiology, University of Tennessee Health Science Center and Le Bonheur Children’s Foundation Research Institute, Memphis, TN USA; 11 Pediatrics and Developmental Neuropsychiatry Branch, National Institute of Mental Health, NIH, Bethesda, MD USA; 12 Unit on Metabolism and Neuroendocrinology, Eunice Kennedy Shriver National Institute of Child Health and Human Development, NIH, Bethesda, MD USA; 13 Department of Psychology, Drexel University, Philadelphia, PA USA; 14 Institute of Psychiatry, King’s College London, London, UK; 15 Cambridgeshire and Peterborough NHS Foundation Trust, Huntingdon, UK

**Keywords:** Cognitive neuroscience, Developmental disorders, Molecular neuroscience

Correction to: *Nature Communications* 10.1038/s41467-020-17051-5, published online 3 July 2020.

The original version of this Article contained an error in the “Results” section, which incorrectly read “For example, the most-extreme ranking CNV region gene relative to anatomical change in each CNV was; +X: *ZCCHC12*, +Y: *EIF1AY*, −X: *GABRA3*, +21: *PCP4*, -22q11: *MAPK1*, -11p13: *TRIM44*.” The correct version states “For example, the most extreme ranking CNV region gene relative to anatomical change in each CNV was: +X: *ZCCHC12*, +Y: *EIF1AY*, −X: *GABRA3*, +21: *PCP4*, -22q11: *SLC7A4*, -11p13: *TRIM44*.”

The original version of this Article contained an error in the “Results” section, which incorrectly read “MS increases in VCFS syndrome (-22q11) and MAPK1 expressing inhibitory neurons.” The correct version removes this sentence.

The original version of this Article contained an error in Figs. 1b and 2d.

In the original version of Fig. 1b, the graph next to the Chr22 schematic reported the following: the *y*-axis range was 0e+00 to 3e−04, the *x*-axis range was −3000 to 3000, and the values for the *P*_RAND-Trans_ and *P*_RAND-Cis_ were 0.0094 and 0.0018, respectively.

The correct version of Fig. 1 is:

which replaces the previous incorrect version:

In the original version of Fig. 2d, the *y*-axis reported the following class of cells: OPC, Oligo, Neuro-In, Endo, Astro. Among the reported genes in the plot, MAPK1 is included.

The correct version of Fig. 2 is:

which replaces the previous incorrect version:

All the errors above have been corrected in both the PDF and HTML versions of the Article.

The original version of the Supplementary Information associated with this Article included an incorrect Supplementary Data 1 file, in which the CVN genes tab, column A (del22q11.2) reported 20 genes in total: DGCR6, PRODH, STK22B, ES2EL, GSCL, SLC25A1, ZDHHC8, RANBP1, HTF9C, DGCR8, T10, ARVCF, COMT, TBX1, GP1BB, UFD1L, HIRA, SNAP29, TMEM191A, MAPK1.

The HTML has been updated to include a corrected version of Supplementary Data 1; the original incorrect version of Supplementary Data 1 can be found as Supplementary Information associated with this Correction.

## Supplementary information

Supplementary Data 1

