# Supplementary material for: Transcriptomic and cellular decoding of regional brain vulnerability to neurogenetic disorders
Source: Nat Commun. 2020 Jul 3;11:3358. doi: 10.1038/s41467-020-17051-5 (PMC7335069; doi:10.1038/s41467-020-17051-5)
Supplement: Supplementary file 1 — Supplementary Information [file 41467_2020_17051_MOESM1_ESM.docx]

**Supplementary Information for:**

**Transcriptomic and Cellular Decoding of Regional Brain Vulnerability to Neurogenetic Disorders**

Jakob Seidlitz^1,2,^*, Ajay Nadig^a^, Siyuan Liu^1^, Richard A.I. Bethlehem^2^, Petra E. Vértes^2,3,4^, Sarah E. Morgan^2^, František Váša^2^, Rafael Romero-Garcia^2^, François M. Lalonde^1^, Liv S. Clasen^1^, Jonathan D. Blumenthal^1^, Casey Paquola^5^, Boris Bernhardt^5^, Konrad Wagstyl^2,6^, Damon Polioudakis^7^, Luis de la Torre-Ubieta^7,8^, Daniel H. Geschwind^7,9^, Joan C. Han^10,11,12^, Nancy R. Lee^13^, Declan G. Murphy^14^, Edward T. Bullmore^2,15^, and Armin Raznahan^1,^*

^1^Developmental Neurogenomics Unit, National Institute of Mental Health, Bethesda, MD, USA.

^2^University of Cambridge, Department of Psychiatry, Cambridge, UK.

^3^School of Mathematical Sciences, Queen Mary University of London, London, UK.

^4^The Alan Turing Institute, London, UK.

^5^McConnell Brain Imaging Centre, Montreal Neurological Institute and Hospital, Montreal, QC, Canada.

^6^McGill Centre for Integrative Neuroscience, McGill University, Montreal, QC, Canada.

^7^Department of Neurology, Center for Autism Research and Treatment, Semel Institute, David Geffen School of Medicine, UCLA, Los Angeles, CA, USA.

^8^Department of Psychiatry and Biobehavioral Sciences, Semel Institute, David Geffen School of Medicine, UCLA, USA.

^9^Department of Human Genetics, David Geffen School of Medicine, UCLA, Los Angeles, CA, USA.

^10^Departments of Pediatrics and Physiology, University of Tennessee Health Science Center and Le Bonheur Children’s Foundation Research Institute, Memphis, TN, USA.

^11^Pediatrics and Developmental Neuropsychiatry Branch, National Institute of Mental Health, NIH, Bethesda, MD, USA.

^12^Unit on Metabolism and Neuroendocrinology, *Eunice Kennedy Shriver* National Institute of Child Health and Human Development, NIH, Bethesda, MD, USA.

^13^Drexel University, Department of Psychology, Philadelphia, PA, USA.

^14^King’s College London, Institute of Psychiatry, London, UK.

^15^Cambridgeshire and Peterborough NHS Foundation Trust, Huntingdon, UK.

* Correspondence to [jakob.seidlitz@nih.gov](mailto:jakob.seidlitz@nih.gov) or [raznahana@mail.nih.gov](mailto:raznahana@mail.nih.gov)

**
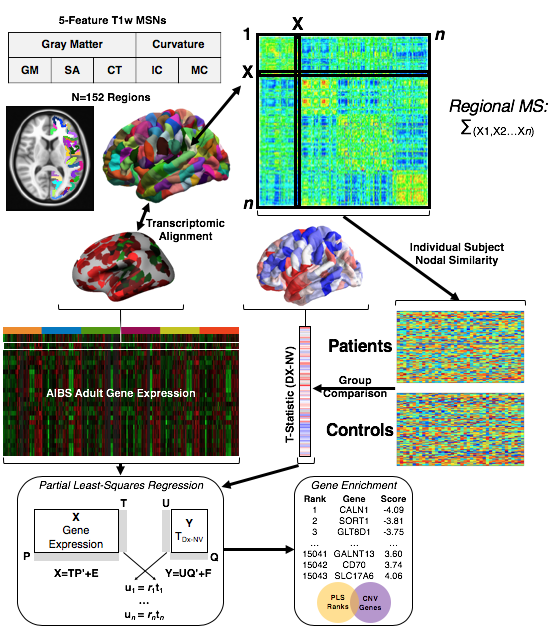
**

**Supplementary Fig. 1. Schematic overview of methodological pipeline.** Morphometric Similarity Networks (MSNs) are constructed for each subject with 5 cortical features derived from structural (T1-weighted) MRI. Regional morphometric similarity (MS) is calculated as the average of unthresholded edges (correlations). Linear regression (‘lm’ function in R) yields T-statistics for CNV effects (patients vs. controls) on each region’s MS estimates. Partial least squares (PLS) regression is performed to generate a ranked list of genes with correlations between regional gene expression and regional MS T-statistics (Methods).

***
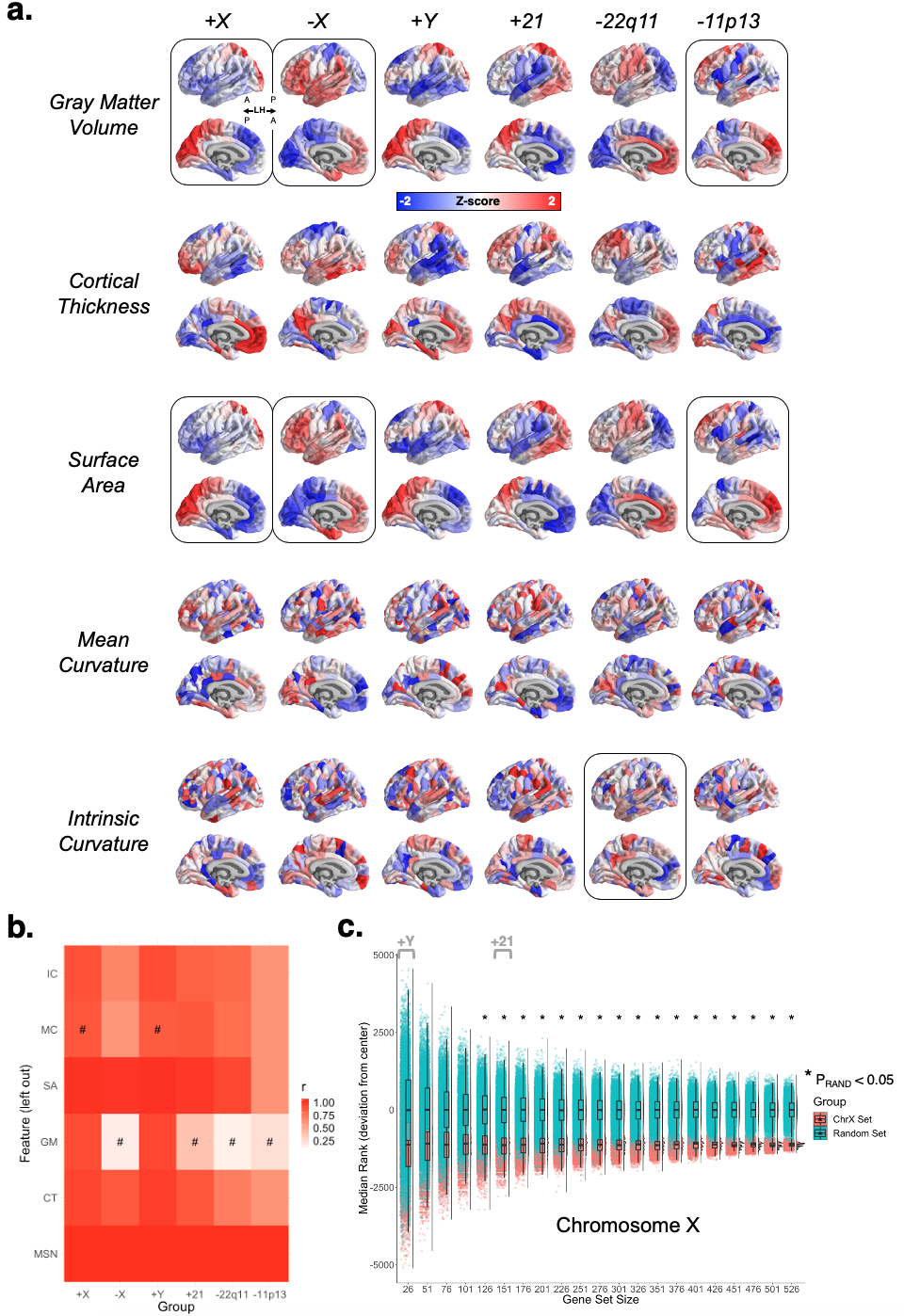
***

**Supplementary Fig. 2. Neuroanatomical effects for individual anatomical features and effects of gene set size on imaging-transcriptomic alignment. a)** Regional T-statistics (CNV patients vs. controls) computed using individual constituent features of the morphometric similarity networks. The CNV gene set ranks linked to these alternative anatomical change maps are provided in Supplementary Dataset 2. Black outlines denote anatomical change maps which successfully recover the preferential spatial coupling between anatomical change and expression of CNV region genes in the human cortex (P_RAND_ < 0.05). **b)** Heatmap showing the correlation between MS change maps computed with all morphological features (MSN) and those with one feature left out. Hashes denote minimum correlations with the original MS change map. **c)** Raincloud plot demonstrating consistency of effect-size between size-varying subsamples of X-chromosome gene ranks for the +X CNV (blue), and random gene subsamples of similar size (red). N of genes per subsample are given on the X-axis. Boxplots show the median, interquartile range (IQR), and whiskers (1.5xIQR). Median ranks are highly consistent, but variation in gene rank increases with reducing gene set size. Black asterisks denote significance of median differences, with a ~100 genes being the smallest gene set size necessary to consistently reach significance at P_RAND_ = 0.05. Gray annotations denote the +Y and +21 CNVs, showing that they fall within the expected trend for significance based on gene sets of similar size within the X-chromosome.

**
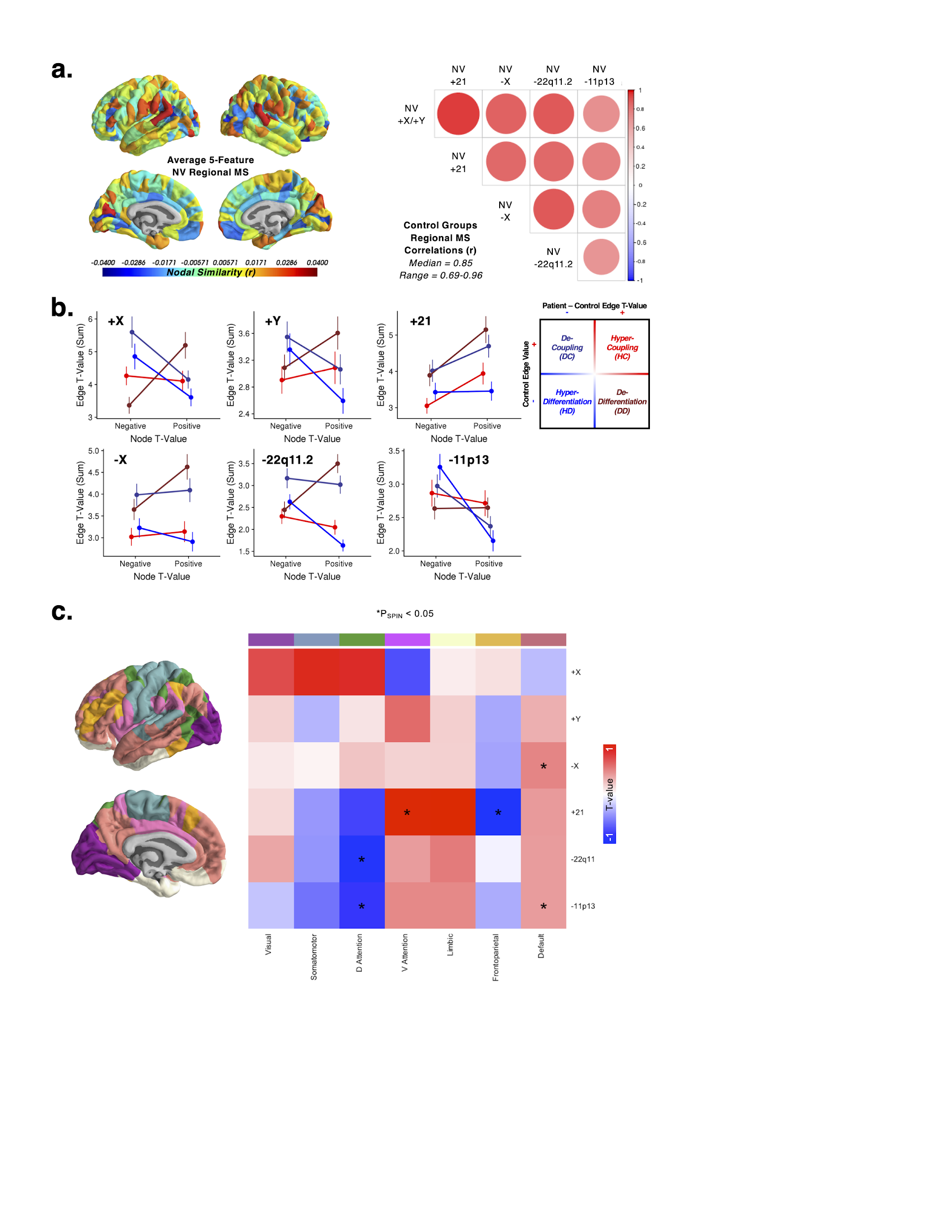
**

**Supplementary Fig. 3. Consistency and interpretation of neuroanatomical effects.** **a)** (Left) Average regional morphometric similarity (MS; mean across edges, or r values) across all 4 independent cohorts of typically-developing controls. There was relatively high regional MS in temporal and parietal regions, and relatively low regional MS in ventromedial prefrontal regions. (Right) Correlation in average regional MS between each set of controls. There was significantly high positive correspondence between the topographies of regional MS (median Pearson’s r = 0.85). **b)** Interpretation of patient-control MS change. Plots show the sum of the absolute T-statistics of CNV effects on MS edges (r values) within the four possible classes of effects (y-axis), for the top 10 most positive and negative regional MS change T-statistics (x-axis). The key shows the four possible outcomes. For each CNV, the regional T-statistics observed (Fig. 1b) arise from a unique combination of the four effects at the edge level. In general, negative regional T-statistics tend to reflect morphometric decoupling in CNV patients relative to controls, whereas positive regional values tend to reflect morphometric de-differentiation in CNV patients relative to controls (see Methods). **c)** Average morphometric similarity change (MS change; T-value) for each of seven canonical functional classes[^17^](https://paperpile.com/c/dbQdSa/XzBET), compared against spatially-permuted MS change maps (P_SPIN_; Methods). NV = normal volunteers. Error bars (pointrange plots) correspond to standard error of the mean.


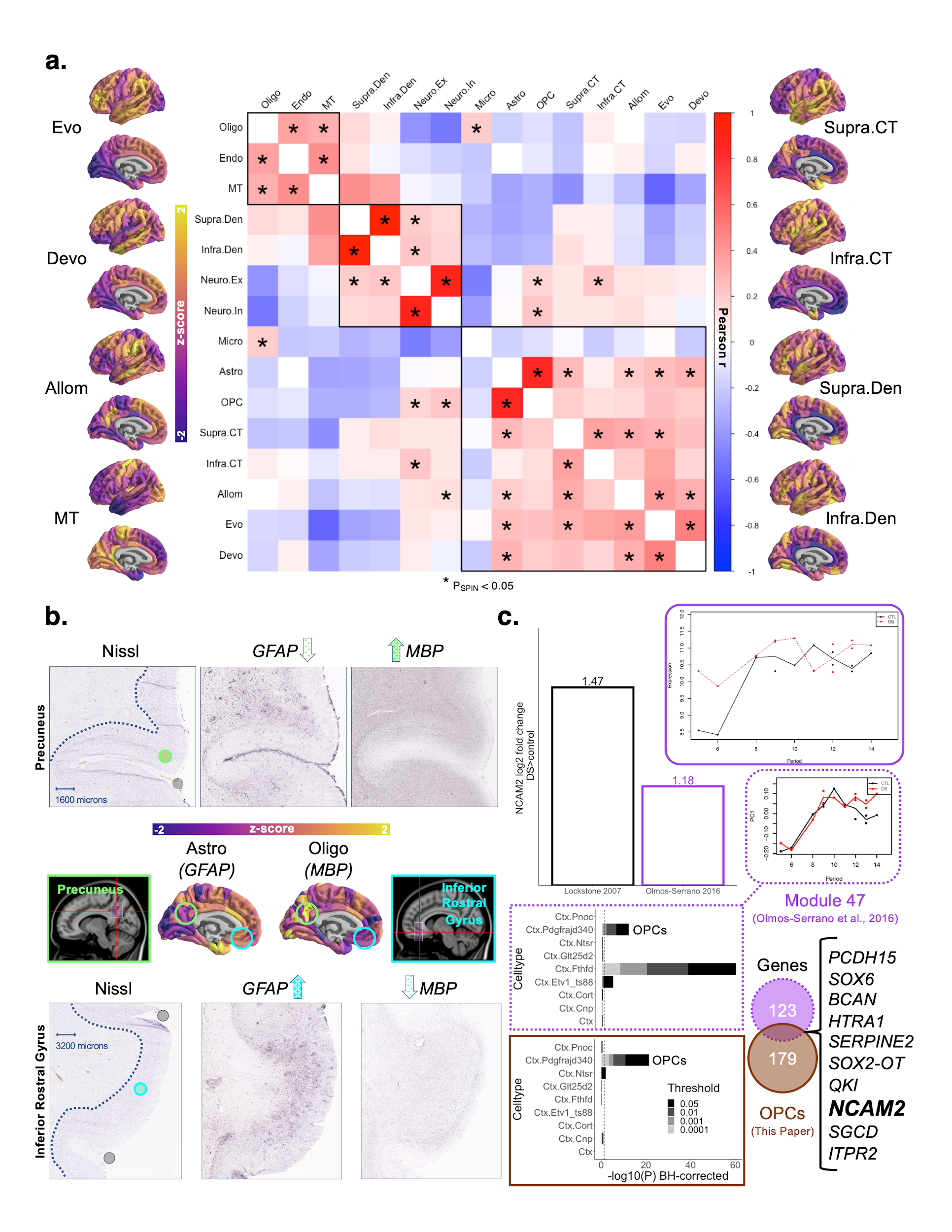


**Supplementary Fig. 4. Validations of cell class expression maps from bulk tissue microarray data and in situ hybridization data.** **a)** Spatial correlations for all map pairs. Asterisks denote P < 0.05 using null models that preserve the spatial contiguity of cortical regions (Methods). Maps are plotted with the same color scale as Fig. 2b. In vivo maps – Evo: evolutionary scaling; Devo: developmental scaling; Allom: allometric scaling; MT: magnetization transfer; From ex vivo (BigBrain) – Supra.CT: supragranular cortical thickness; Infra.CT: infragranular cortical thickness; Supra.Den: supragranular density; Infra.Den: infragranular density. **b)** Double-dissociation validation of cell-specific expression using in situ hybridization (ISH). Non-normalized slice images show the nearest Nissl reference slice and staining for two canonical marker genes: *GFAP* for astrocytes (Astro) and *MBP* for oligodendrocytes (Oligo). This data came from the right hemisphere of donor H0351.1009, one of the six donors for the bulk transcriptomic dataset and whose left hemisphere was included in the transcriptomic mapping (Methods). Sagittal slices depict the approximate location of corresponding ISH slab on the MNI152 template. Dotted blue line denotes manually-drawn white matter/gray matter boundary. **c)** Barplot of *NCAM2* differential expression (y-axis fold-change) between Down syndrome (DS) patients and controls (CTL) from two independent studies (on x-axis) using bulk sequencing from brain tissue[^28,30^](https://paperpile.com/c/dbQdSa/0h2dj+cwNEw). Solid purple-outlined plot shows *NCAM2* expression in DS patients relative to CTL across developmental windows. Dashed purple-outlined plot shows Module 47 expression[^28,30^](https://paperpile.com/c/dbQdSa/0h2dj+cwNEw) across the same windows. This module contained *NCAM2*, and was found to be highly enriched for oligodendrocyte progenitor cells (OPCs; see Figure 2). (bottom) Independent verification of OPC enrichment for the Module 47 gene set[^28,30^](https://paperpile.com/c/dbQdSa/0h2dj+cwNEw) (N=123), as well as the combined OPC gene list (from the current paper), using Cell-Specific Enrichment Analysis[^69^](https://paperpile.com/c/dbQdSa/eY2SI) (CSEA). The overlapping gene set contained the gene of interest, *NCAM2*, identified by the analyses in Fig2d. Ctx: cortex; PDgfrajd340: Oligodendrocyte Progenitor Cells; Etv1_ts88: Immune Cells; Pnoc: Pnoc+ neurons; Ntsr: Ntsr+ neurons; Glt25d2: Glt25d2 neurons; Fthfd: Astrocytes; Cort: Cort+ neurons; Cnp: Myelinating Oligodendrocytes.

**
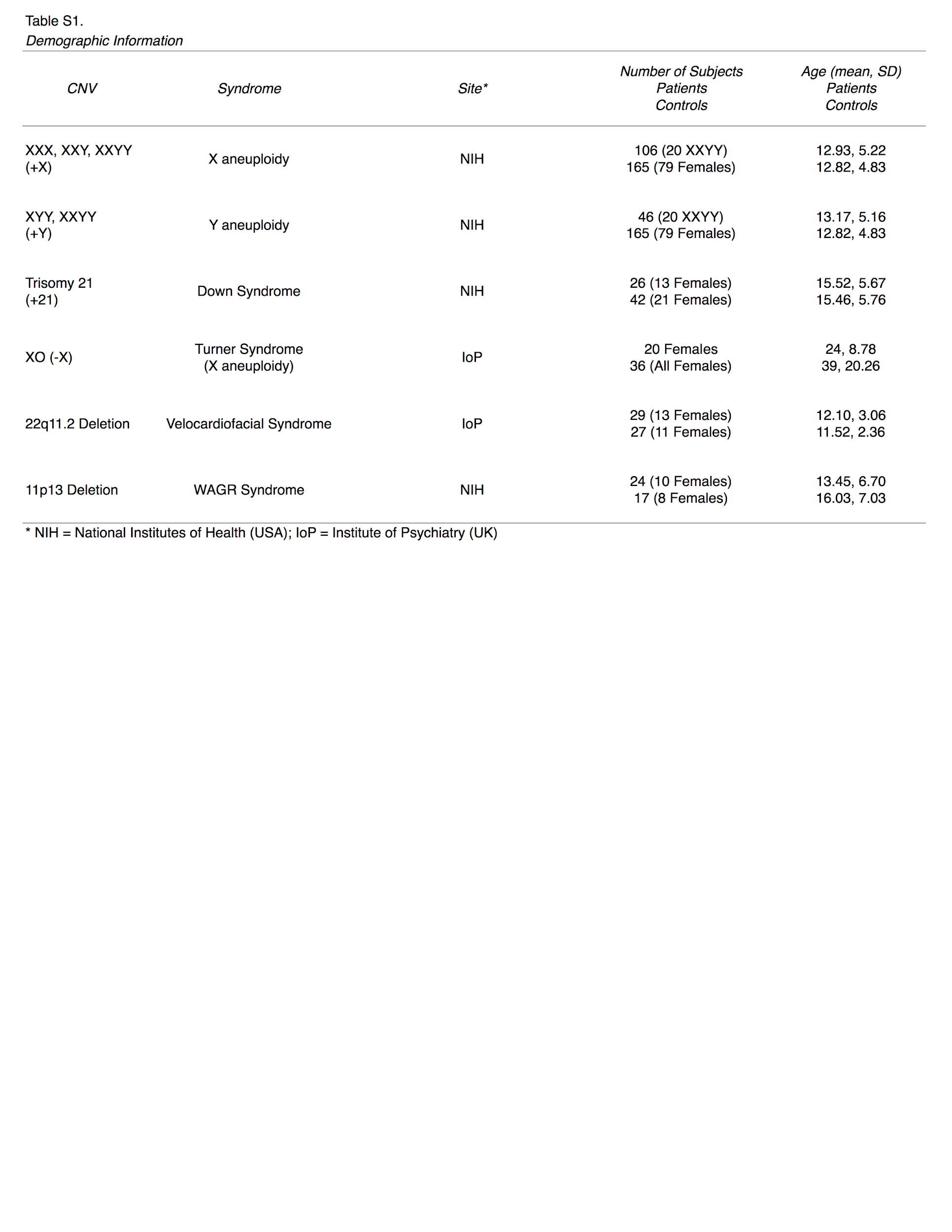
**

**Supplementary Table 1.** Participant Characteristics.

**Supplementary Dataset 1.** CNV Gene set median ranks for MS change maps.

**Supplementary Dataset 2.** CNV Gene set median ranks for anatomical change maps for individual cortical features.

**Supplementary Dataset 3.** CNV genes. Ranked gene lists for each CNV from PLS analysis.

**Supplementary Dataset 4.** GO annotations for ranked gene lists.

**Supplementary Dataset 5.** Cell-type gene sets.

**Supplementary Dataset 6.** Dosage sensitive gene sets.

**Supplementary Dataset 7.** List of brain-expressed genes.
