# Supplementary material for: Transcriptomic and cellular decoding of regional brain vulnerability to neurogenetic disorders
Source: Nat Commun. 2020 Jul 3;11:3358. doi: 10.1038/s41467-020-17051-5 (PMC7335069; doi:10.1038/s41467-020-17051-5)
Supplement: Supplementary file 3 — Description of Additional Supplementary Files [file 41467_2020_17051_MOESM3_ESM.pdf]

Description of Additional Supplementary Files

Title: Supplementary Data 1.

Description: CNV Gene set median ranks for MS change maps.

Title: Supplementary Data 2.

Description: CNV Gene set median ranks for anatomical change maps for individual cortical features.

Title: Supplementary Data 3.

Description: CNV genes. Ranked gene lists for each CNV from PLS analysis.

Title: Supplementary Data 4.

Description: GO annotations for ranked gene lists.

Title: Supplementary Data 5.

Description: Cell-type gene sets.

Title: Supplementary Data 6.

Description: Dosage sensitive gene sets.

Title: Supplementary Data 7.

Description: List of brain-expressed genes.
