# Supplementary material for: Transcriptomic and cellular decoding of regional brain vulnerability to neurogenetic disorders
Source: Nat Commun. 2020 Jul 3;11:3358. doi: 10.1038/s41467-020-17051-5 (PMC7335069; doi:10.1038/s41467-020-17051-5)
Supplement: Supplementary file 11 — Reporting Summary [file 41467_2020_17051_MOESM11_ESM.pdf]

nature research

Corresponding author(s): Jakob Seidlitz; Armin Raznahan

Last updated by author(s): Apr 29, 2020

Reporting Summary

Nature Research wishes to improve the reproducibility of the work that we publish. This form provides structure for consistency and transparency in reporting. For further information on Nature Research policies, see Authors & Referees and the Editorial Policy Checklist .

Statistics

For all statistical analyses, confirm that the following items are present in the figure legend, table legend, main text, or Methods section.

n/a	Confirmed	
☐	☒	The exact sample size (n) for each experimental group/condition, given as a discrete number and unit of measurement
☐	☒	A statement on whether measurements were taken from distinct samples or whether the same sample was measured repeatedly
☐	☒	The statistical test(s) used AND whether they are one- or two-sided<br>*Only common tests should be described solely by name; describe more complex techniques in the Methods section.*
☐	☒	A description of all covariates tested
☐	☒	A description of any assumptions or corrections, such as tests of normality and adjustment for multiple comparisons
☐	☒	A full description of the statistical parameters including central tendency (e.g. means) or other basic estimates (e.g. regression coefficient) AND variation (e.g. standard deviation) or associated estimates of uncertainty (e.g. confidence intervals)
☐	☒	For null hypothesis testing, the test statistic (e.g. F, t, r) with confidence intervals, effect sizes, degrees of freedom and P value noted<br>*Give P values as exact values whenever suitable.*
☒	☐	For Bayesian analysis, information on the choice of priors and Markov chain Monte Carlo settings
☒	☐	For hierarchical and complex designs, identification of the appropriate level for tests and full reporting of outcomes
☐	☒	Estimates of effect sizes (e.g. Cohen's d, Pearson's r), indicating how they were calculated

Our web collection on statistics for biologists contains articles on many of the points above.

Software and code

Policy information about availability of computer code

Data collection	Multiple MRI scanners (see Methods)
Data analysis	CIVET1.1.10 and Caret5 were used for preprocessing of the neuroimaging data. RStudio1.0.153 was used for statistical analyses. RStudio1.0.153 and Python2.7 were for plotting, BrainsForPublication v0.2.1 for cortical surface representations, gene expression mapping: https://github.com/RafaelRomeroGarcia/geneExpression_Repository, and rotation permutation method: https://github.com/frantisekvasa/rotate_parcellation

For manuscripts utilizing custom algorithms or software that are central to the research but not yet described in published literature, software must be made available to editors/reviewers. We strongly encourage code deposition in a community repository (e.g. GitHub). See the Nature Research guidelines for submitting code & software for further information.

Data

Policy information about availability of data

All manuscripts must include a data availability statement. This statement should provide the following information, where applicable:

- Accession codes, unique identifiers, or web links for publicly available datasets
- A list of figures that have associated raw data
- A description of any restrictions on data availability

Relevant code and processed data (i.e., Extended Data Tables and brain maps) for generating all findings and figures presented in this manuscript will be made available on Github: https://github.com/jms290/PolySyn_MSNs

Field-specific reporting

Please select the one below that is the best fit for your research. If you are not sure, read the appropriate sections before making your selection.

☒ Life sciences ☐ Behavioural & social sciences ☐ Ecological, evolutionary & environmental sciences

For a reference copy of the document with all sections, see nature.com/documents/nr-reporting-summary-flat.pdf

Life sciences study design

All studies must disclose on these points even when the disclosure is negative.

Sample size	Sample size was determined by aggregating patient/control datasets from previous studies to generate the largest possible overall sample size of neurogenetic syndromes. Due to the rare incidence of the patient cohorts, each patient cohort size included an age and sex matched independent control cohort for (also see "Replication"). References for these studies can be found in the Methods section of the manuscript.
Data exclusions	Criteria were pre-established from previous work for inclusions of data, which included exclusion based on quality control of the structural MRI scans as well as the processed cortical morphometrics and surface reconstructions. Further verification (visual quality control) of these inclusions was performed/confirmed for the neuroimaging data in all cohorts.
Replication	6 independent patient and matched control groups were included. Additionally, each independent control cohort was assessed for stability in our neuroimaging methods (Supplementary Figure 3).
Randomization	Experimental groups were pre-determined based on categorical diagnosis for each neurogenetic syndrome. In our neuroimaging analyses, we include sex and age as covariates when assessing for patient effects on neuroanatomy. To robustly estimate p values in our neuroimaging-transcriptomic analyses, we employ multiple randomization (permutation) strategies, taking into account space (brain anatomy), and set (gene set size and location for the imaging-transcriptomic annotations). See Methods for additional details on these procedures.
Blinding	No blinding was performed. All group assignments were known based on patient/control status such that hypothesis testing could be performed in the neuroimaging-transcriptomic analyses (see Figure 1).

Reporting for specific materials, systems and methods

We require information from authors about some types of materials, experimental systems and methods used in many studies. Here, indicate whether each material, system or method listed is relevant to your study. If you are not sure if a list item applies to your research, read the appropriate section before selecting a response.

Materials & experimental systems

n/a	Involved in the study	
☒	☐	Antibodies
☒	☐	Eukaryotic cell lines
☒	☐	Palaeontology
☒	☐	Animals and other organisms
☐	☒	Human research participants
☒	☐	Clinical data

Methods

n/a	Involved in the study	
☒	☐	ChIP-seq
☒	☐	Flow cytometry
☐	☒	MRI-based neuroimaging

Human research participants

Policy information about studies involving human research participants

Population characteristics	All information can be found in Supplementary Table 1 and the Methods section
Recruitment	Patient cohorts were independently recruited based on the clinical protocols enumerated in the main manuscript (Methods section), which included community-based and clinical strategies.
Ethics oversight	Each independent cohort's data collection was approved by the relevant governing body. Statements can be found in the Methods.

Note that full information on the approval of the study protocol must also be provided in the manuscript.

October 2018

Magnetic resonance imaging

Experimental design

Design type	structural MRI acquisition
Design specifications	1 scan per subject
Behavioral performance measures	The current study focused on neuroanatomical measurements derived from structural MRI where behavioral performance measures were not relevant.

Acquisition

Imaging type(s)	T1-weighted structural MRI
Field strength	1.5 and 3
Sequence & imaging parameters	see Methods for specific parameters for each cohort
Area of acquisition	whole brain

Diffusion MRI ☐ Used ☒ Not used

Preprocessing

Preprocessing software	were performed using the algorithms employed by the CIVET (v1.1.10) software package.
Normalization	linear and nonlinear deformations to the template were performed using the algorithms employed by the CIVET (v.1.1.10) software package.
Normalization template	ICBM152 ("MNI space")
Noise and artifact removal	Human-determined quality assessments
Volume censoring	A single volume was collected for each subject, and therefore censoring was equivalent to quality control, as outlined above and in the main manuscript.

Statistical modeling & inference

Model type and settings	Standard linear models were run for each region across subjects to determine T-statistics for disease effects on brain anatomy. Covariates are described in the Methods for each cohort. These continuous T-statistic maps were carried forward in the imaging-transcriptomic analyses, where multivariate methods (PLS regression) was employed
Effect(s) tested	see previous point

Specify type of analysis: ☒ Whole brain ☐ ROI-based ☐ Both

Statistic type for inference (See Eklund et al. 2016)	Whole-brain parcellation continuous values were carried forward to imaging-transcriptomic analyses
Correction	See Methods and above for descriptions on the multiple permutation strategies employed to test robustness of empirical effects

Models & analysis

n/a	Involved in the study
☒	☐ Functional and/or effective connectivity
☒	☐ Graph analysis
☐	☒ Multivariate modeling or predictive analysis

Multivariate modeling and predictive analysis	Partial Least Squares (PLS) regression was used in two contexts (i) for ranking genes based on their spatial relationship to brain maps (common dimension = brain regions), and (ii) to assess interindividual variation in gene expression based on interindividual variation in brain maps (common dimension = person). Resampling and spatial permutation (see Methods) was used to determine P values for fits of the PLS model.
